# Skyshine photon doses from 6 and 10 MV medical linear accelerators

**DOI:** 10.1120/jacmp.v13i1.3671

**Published:** 2012-01-05

**Authors:** Eduardo de Paiva, Luiz A. R. da Rosa

**Affiliations:** ^1^ Instituto de Radioproteção e Dosimetria – IRD/CNEN/MCT, Recreio dos Bandeirantes Rio de Janeiro – RJ Brazil

**Keywords:** skyshine, shielding, medical accelerator

## Abstract

The skyshine radiation phenomenon consists of the scattering of primary photon beams in the atmosphere above the roof of a medical linear accelerator facility, generating an additional dose at ground level in the vicinity of the treatment room. Thus, with respect to radioprotection, this situation plays an important role when the roof is designed with little shielding and there are buildings next to the radiotherapy treatment room. In literature, there are few reported skyshine‐measured doses and these contain poor agreement with empirical calculations. In this work, we carried out measurements of skyshine photon dose rates produced from eight different 6 and 10 MV medical accelerators. Each measurement was performed outside the room facility, with the beam positioned in the upward direction, at a horizontal distance from the target and for a 40 cm×40 cm maximum photon field size at the accelerator isocenter. Measured dose‐equivalent rates results were compared with calculations obtained by an empirical expression, and differences between them deviated in one or more order of magnitude.

PACS numbers: 87.53.‐j, 87.53.Bn

## I. INTRODUCTION

When the air above the roof of a radiotherapy facility room scatters the primary photon beam from a medical linear accelerator, this stray radiation is known as skyshine radiation.^(^ ^1^ ^)^ The skyshine doses are particularly important when the ceiling of a teletherapy treatment room is designed with little or no shielding.

There are few reported experiments on secondary skyshine radiation,^(^ ^2^ ^,^ ^3^ ^)^ so new measurements of this type of radiation play an important role on the understanding of the skyshine phenomenon itself. The aim of this work is to compare the measured skyshine photon dose rates in the vicinity of eight different teletherapy treatment rooms housing 6 MV and 10 MV medical accelerators with empirical calculations as previously reported.^(^ ^2^ ^,^ ^3^ ^)^ Consideration of the leakage radiation from the accelerator head transmitted through the lateral wall to the measurements of skyshine dose rate is also discussed.

## II. MATERIALS AND METHODS

**Figure 1:**
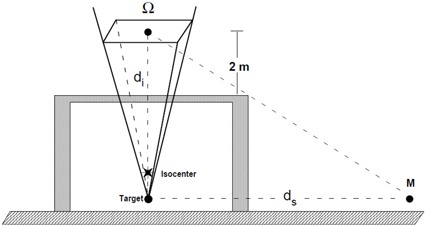
The geometry used in skyshine photon dose rates measurements in the vicinity of radiotherapy treatment rooms.

The geometry employed in skyshine photon dose rates measurements carried out in this work is shown in Fig. 1. The photon beam is directed upward (180° gantry position), with a maximum 40 cm×40 cm photon field size at isocenter and with no phantom. The photon beam forms a solid angle Ω defined as:^(^ ^4^ ^)^

(1) $$\Omega =4\;\text{arcsin}\frac{a^{2}}{a^{2}+4h^{2}}$$

which depends on the field size *a* (40 cm) and the source‐axis distance *h* (100 cm). As pointed out in the work of Gossman et al.,^(^ ^4^ ^)^ Eq. (1) gives the correct way to determine the solid angle when the radiation beam resembles an inverted pyramid of base a × a.

The term $d_{i}$ that appears in Fig. 1, is the distance in meters from the target to a point 2 meters above the roof, and $d_{s}$ is the horizontal distance in meters from the isocenter to the point M where the dose rate is measured.

The survey meters used in the measurements of equivalent dose rate $\overset{•}{H}$ were ionization chambers, namely SmartION models 2120 R, serial numbers 4628 and 4779 (Thermo Fisher Scientific, Wiltham, MA) and Inovision models 451P, serial numbers 541 and 542 (Inovision Software Solutions Inc., Chesterfield, MI), all of them annually calibrated in the Secondary Standard Dosimetry Laboratory (SSDL) of Brazil. The uncertainty associated with survey measurements was provided by the calibration procedure and was better than 10% for a confidence level of 95%.

In this study, the data of skyshine radiation measurements from eight different medical linear accelerators were analyzed. The equipments were seven 6 MV machines and one 10 MV accelerator, produced by Varian Medical Systems and Siemens Medical Solutions Manufacturers. They were installed in different radiotherapy facilities. The points M (1.5 m above the ground surface) where measurements were conducted were localized in uncontrolled areas with integral occupancy, namely parking lots or gardens. For each facility, the dose rate was measured only at the point M, shown in Fig. 1, which is the nearest point located outside the treatment room that an observer *hypothetically* located two meters above the ceiling in central axis of the beam can see.

Leakage radiation can traverse the lateral wall of the treatment room and also reach the location of measurements contaminating the skyshine radiation level. So the leakage dose rate should be subtracted from the total dose rate at M. The leakage contribution to the dose rate can be evaluated by setting up a 0 cm × 0 cm field size, when no radiation scattered on the air above the roof exists. Another contribution to the dose rate at point M is the primary radiation scattered on the inside walls of the room, but we assumed it to be negligible.

In previous studies,^(^ ^2^ ^,^ ^3^ ^,^ ^5^ ^)^ the following empirical expression was used to calculate the dose‐equivalent rate H (in nSv/h) due to skyshine radiation:

(2) $$\dot{H}=\frac{2.5\times 10^{7}\left(B.{\dot{D}}_{0}.\Omega^{1.3}\right)}{\left(d_{i}\;.\;d_{s}\right)}$$

where ${\dot{D}}_{0}$ is the X‐ray absorbed dose output rate (in Gy/h) at the accelerator isocenter and B is the transmission coefficient through the roof (which in all cases investigated in this study were made of common concrete of $2.35\;g/{\text{cm}}^{3}$ density) and can be calculated by:

(3) $$B=10^{-\left\{1+\left[\frac{\left(t-TVL_{1}\right)}{TVL_{e}}\right]\right\}}$$

In the equation above, ${\text{TVL}}_{1}$ and ${\text{TVL}}_{e}$ are the first and second tenth‐value layers of common concrete, which are, respectively, 37 cm and 33 cm for an endpoint energy of 6 MV, and 41 cm and 37 cm for an endpoint energy of 10 MV,^(^ ^5^ ^)^ and t is the barrier thickness. Taking into consideration the thicknesses of the ceiling of the treatment rooms analyzed in this work, we obtained the transmission coefficient B in the range 0.001–0.081.

The X‐ray absorbed dose output rates for the eight accelerators ranged from 200 to 250 cGy/min at isocenter. The vertical distance $d_{i}$ was ≈ 5.5 meters for the rooms at all facilities, and the horizontal distance $d_{s}$ was in the 8.8 m–15 m range.

Following the generally accepted recommendation to always use the worst situation in the calculation of barrier shielding (i.e., the largest doses which imply higher barrier thicknesses), no phantom was placed crossing the radiation pathway and a maximum 40 cm×40 cm field size was used along all measurements. Therefore, the solid angle remained constant and equal to 0.1539 steradians.

## III. RESULTS & DISCUSSION

**Figure 2:**
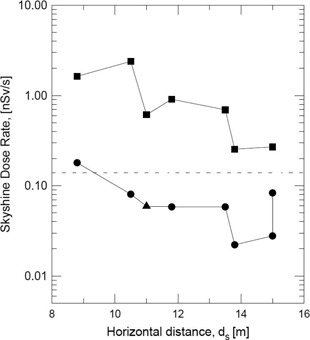
The equivalent dose rate as a function of the horizontal distance from isocenter. Circles=6 MV MV measured dose rates; triangle=10 MV measured dose rate; squares=calculations by means of Eq. (2); dashed line represents the permissible dose rate limit for uncontrolled areas.

The measurements of skyshine radiation level at point M outside the treatment room were performed according to the geometry depicted in Fig. 1. The measured equivalent‐dose rate H as a function of the horizontal distance $d_{s}$ from the isocenter for eight different medical linear accelerators is showed in Fig. 2. In Fig. 2, the error bars assigned to the calibration procedure cannot be seen because they are smaller than the symbols and the full lines are merely to guide the eyes. In Fig. 2, circles stand for 6 MV measured dose rates, triangle stands for 10 MV measured dose rate, and squares stand for empirical calculations.^(^ ^2^ ^,^ ^3^ ^,^ ^5^ ^)^ Dashed line represents the permissible dose rate limit for uncontrolled areas.^(^ ^6^ ^)^

In the measurements carried out in this simple work we intended to use always the worst situation in the assessment of the photon dose rate due to skyshine. This conservative assumption permitted us to set up the unchanged 40 cm×40 cm photon beam field size along the measurements.

In our investigation, we also did not consider horizontal distances smaller than the distance $d_{s}$ showed in Fig. 1. These points are inside a shadow cone where the dose rates are expected to be lower, a result already observed in the work by Gossman et al.^(^ ^3^ ^)^

In this study, we also investigated the dose rate due to leakage radiation from the accelerator head that is transmitted through the lateral wall of the treatment room and can be summed up to the skyshine dose rate at point of interest. This leakage contribution was evaluated by setting up a 0 cm × 0 cm field size at isocenter when no skyshine contribution exists. Measurements have indicated that leakage component to dose at M represents ≤ 1% of the total dose.

As can be observed in Fig. 2, the majority of measured dose rates data proved to be no greater than the value 0.02 mSv/week (or 0.139 nSv/sec) of the permissible dose limit for uncontrolled areas.^(^ ^5^ ^,^ ^6^ ^)^ In Fig. 2, we also show empirical calculations obtained by means of Eq. (2) and using the parameters described in the previous section. In general, these estimations resulted in values one order of magnitude or more above the measured ones at distances from the lateral wall up to the point M shown in Fig. 1, a difference already reported in other works.^(^ ^2^ ^,^ ^3^ ^)^

## IV. CONCLUSIONS

The largest photon field sizes, which correspond to the highest doses, were used to investigate the contribution of the secondary skyshine radiation to the dose rates outside teletherapy treatment rooms housing 6 and 10 MV linear accelerators. Measurements have indicated that only one value of skyshine dose rate was greater than the permissible shielding design goal for the uncontrolled areas investigated, due to poor shielding in that room. A poor agreement, of one order of magnitude or more, was found between measured skyshine photon dose rates and empirical estimations, indicating that such a calculations should be used with caution and serve only as a rough estimation of skyshine dose rates contribution. Apart from the large discrepancies found between calculations and measurements of photon dose rate, empirical calculations have the merit of showing the dependency between skyshine radiation and treatment room geometric quantities.
